# The Effect of a Ketogenic Low-Carbohydrate, High-Fat Diet on Aerobic Capacity and Exercise Performance in Endurance Athletes: A Systematic Review and Meta-Analysis

**DOI:** 10.3390/nu13082896

**Published:** 2021-08-23

**Authors:** Jingguo Cao, Siman Lei, Xiuqiang Wang, Sulin Cheng

**Affiliations:** 1 Exercise, Health and Technology Centre, Department of Physical Education, Shanghai Jiao Tong University, Shanghai 200240, China; 370321199703212716@sjtu.edu.cn (J.C.); alicelei@um.edu.mo (S.L.); sulin.cheng@jyu.fi (S.C.); 2 Faculty of Education, University of Macau, Macau SAR, China; 3 Exercise Translational Medicine Centre, Shanghai Centre for Systems Biomedicine, Shanghai Jiao Tong University, Shanghai 200240, China; 4 Faculty of Sport and Health Science, University of Jyväskylä, 40014 Jyväskylä, Finland

**Keywords:** ketogenic low-carbohydrate, high-fat diet, aerobic capacity, endurance athletes

## Abstract

A low-carbohydrate, high-fat (LCHF) diet has been proposed to enhance the fat utilization of muscle and the aerobic capacity of endurance athletes, thereby improving their exercise performance. However, it remains uncertain how the macronutrient intake shift from carbohydrate to fat affects endurance exercise training and performance. This study performed a systematic review and meta-analysis to explore the effects of a ketogenic low-carbohydrate, high-fat (K-LCHF) diet on aerobic capacity and exercise performance among endurance athletes. Searches were carried out in five electronic databases, and we followed the Preferred Reporting Items for Systematic Review and Meta-Analyses (PRISMA) guidelines. The search included studies using an LCHF diet as an intervention protocol and compared data on factors such as maximum oxygen uptake (VO_2_max) and rating of perceived exertion (RPE) from the graded exercise test. In this case, 10 studies met the criteria and were included in the meta-analysis. We did not find a significant effect of K-LCHF diet interventions on VO_2_max, time to exhaustion, HRmax or RPE. However, a significant overall effect in the substrate oxidation response to respiratory exchange rate was observed. The meta-analysis showed that K-LCHF diets did not affect aerobic capacity and exercise performance. Therefore, high-quality interventions of a K-LCHF diet are needed to illustrate its effect on various endurance training programs.

## 1. Introduction

The low-carbohydrate, high-fat (LCHF) diet has become popular as a treatment for excess weight, epilepsy and type 2 diabetes in recent decades [1,2,3]. The first LCHF diet study to optimize fat oxidation in endurance athletes was conducted in 1983 by Phinney et al. [4]. He spotlighted various mechanisms to boost endurance performance by promoting a shift in substrate utilization to enhance physiological training benefits by adopting the LCHF diet. However, the concept of improving athletic performance by adapting to a high-fat diet was reconsidered after a series of studies failed to prove significant benefits [5]. As a result, the pace of research in this area has slowed significantly. However, with the resurgence in popularity of “Paleolithic” and “ketogenic” diets, there has been renewed interest in the LCHF diet [6].

Limiting carbohydrate (CHO) consumption can reduce the muscle glycogen concentration, resulting in greater fat oxidation [7,8]. After adapting to the LCHF diet, the body uses more fat for energy, and fat stores are far more abundant than CHO, thus theoretically providing energy for a longer period [9]. A ketogenic LCHF (K-LCHF) diet may influence the adaptation of the body through the molecular mechanism of regulating cell signal transduction [10,11,12,13]. In addition, this signaling pathway’s activation may lead to increased physical and motor ability through, for example, mitochondrial biogenesis, capillary thinning and regeneration processes, especially the efficient utilization of fat energy substrates [14,15,16]. Of note, in the study on adopting ketogenic diets in mice, they found that long-term ketogenic diet might decreased mitochondrial biogenesis, impaired cellular respiration and increased myocardial apoptosis and myocardial fibrosis [17]. The energy obtained from fat under aerobic conditions produces acetoacetic acid, β-hydroxybutyric acid (β-HB) and acetone, of which β-HB accounts for about 70% of the ketone body, which serves as a stable energy source for the body and brain [17,18].

The review by Hawley et al. was the first to summarize the effect of the LCHF diet on exercise performance and metabolism. He found that long-term (>7-day) use of the LCHF diet extended endurance for a fixed, sub-maximum workload in well-trained athletes [19]. One study from Burke examined the LCHF diet’s acute effect and found that it took just five days for muscles to adapt [20]. The metabolic adaptations needed for the full benefit produced from adaptation to LCHF diets are suggested by the long time it takes to lower the rate of carbohydrate oxidation and glycogen utilization [21]. However, LCHF diets have been shown to have mixed results [22], with some studies reporting positive effects [23,24], while other studies finding that prolonged adaptation might not change performance [25,26]. A relatively long period on the LCHF diet did not affect performance in endurance exercise and resistance training [27,28]. In addition, individuals might have different adaptation processes to the LCHF diet [29]. Studies have suggested that, beyond diet duration, other variables may influence the effect of the LCHF diet on exercise performance (e.g., training status, performance test type, intensity and sex differences [30]).

The ketogenic diet is a special case of an LCHF diet. Some studies have suggested that LCHF diets with CHOs accounting for less than 5% [31] or between 5% and 10% [32] of total energy intake belong to the ketogenic diet. Furthermore, it is proposed that diets with less than 10% CHOs can induce ketosis [33]. As there is overlap in the definitions of the LCHF and ketogenic diets, most studies use the term “LCHF”, some use the term “keto” and some use “K-LCHF”, even though the content of these diets are similar. In this study, we use K-LCHF [30] to indicate the diet intervention.

Recent studies on the K-LCHF diet have not systematically analyzed the effects of the K-LCHF diet on endurance performance and related indicators. The effects of the K-LCHF diet on athletes during endurance exercise are controversial. Hence, the objective of this systematic review and meta-analysis was to aggregate the results from experimental data to investigate the overall effects of the K-LCHF diet on aerobic capacity and exercise performance in endurance athletes.

## 2. Materials and Methods

### 2.1. Literature Search

By following the Preferred Reporting Items for Systematic Review and Meta-Analyses (PRISMA) guidelines, we systematically searched online databases for studies published until April 2021. The study protocol was registered in the international database of prospectively registered systematic reviews in health and social care (PROSPERO: 226008). The literature search identified full-text articles published in peer-reviewed scientific journals in English from five online databases: Ovid Web of Science, PubMed, Science Direct, ProQuest and Cochrane library. The search strategy (Table S2) was conducted independently by two authors (J.C. and S.L.). When conflicting opinions arose, the eligibility of the studies was discussed with the other authors (S.C and X.W.) until an agreement was reached. The search strategy and keywords/terms included “low-carbohydrate high-fat diet” or “ketogenic diet” and “aerobic capacity” or “athlete” or “VO_2_max” or “exercise” or “training”. Additional studies were retrieved by examining the reference lists of the relevant articles. Reference lists from previous relevant reviews and included studies were further examined as complementary sources.

### 2.2. Inclusion and Exclusion Criteria

The screening process was divided into two phases: a preliminary selection by title and abstract only, and a second phase of screening the full text of the remaining articles. Articles that met the following criteria were included: (1) randomized controlled intervention trial or controlled intervention trial or crossover trial in endurance athletes (including professional athletes and individuals who participated in endurance exercise as their hobby); (2) a LCHF diet with less than 10% CHOs is considered a K-LCHF diet [31,32]; (3) comparison of the effects of the K-LCHF diet with those of the non-LCHF diet; (4) performed graded exercise test (GXT); 5) reported daily nutrition intake based on food diaries or recalls; (5) endurance training was defined as long-duration continuous sustain moderate or submaximal intensity exercise of 5 min to 4 h [34,35].

The exclusion criteria included (1) systematic reviews or meta-analyses, (2) observational studies, (3) no appropriate control group and (4) non-endurance athletes.

### 2.3. Data Extraction

Data extracted from the studies included number of subjects, age, gender, study design, intervention duration, dietary components, energy intake, type of exercise, maximal oxygen uptake (VO_2_max), time to exhaustion (TTE), maximal heart rate during exercise (HRmax), respiratory exchange rate (RER) and rating of perceived exertion (RPE). The authors, publication year, study design, outcome variables, testing protocol for outcomes, participant characteristics and exercise protocol were extracted and are summarized in Table 1. Baseline and post-intervention outcome variables were taken from all intervention and control groups as the mean and standard deviation (SD), as recommended by the *Cochrane Collaboration Handbook* [36]. If the mean or SD was reported in the original article, the established methods were used [36,37]; otherwise, the original data were obtained by contacting the study authors directly.

VO_2_max indicates the aerobic capacity, the VO_2_max test [38] refers to the aerobic capacity measurement, and TTE represents the endurance exercise performance [39] in a laboratory environment.

### 2.4. Risk of Bias and Quality Assessment

**Figure 1:**
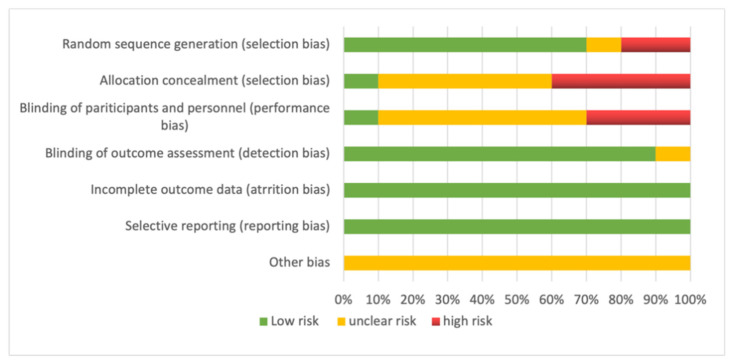
Risk of bias included studies in meta-analysis.

Two of the authors (J.C. and S.L.) assessed the risk of bias using Review Manager software (RevMan, version 5.4, Cochrane Collaboration, Oxford, UK), which is summarized as a risk of bias in Figure 1. The validity of the studies was assessed using the Cochrane Collaboration risk-of-bias tool. This tool includes the following items: “Random sequence generation (selection bias)”, “Concealment of the allocation sequence (selection bias)”, “Blinding of participants and personnel (performance bias)”, “Blinding of outcome assessment (detection bias)”, “Incomplete outcome data (attrition bias)”, “Selective outcome reporting (reporting bias)” and “Other biases”, which were assigned as “low risk of bias”, “unclear risk of bias” or “high risk of bias”.

The quality of the included articles was evaluated using the Assessment of Multiple Systematic Review (AMSTAR) measurement tool [40] and is presented in Table S1. This tool has 11 items (i.e., “A prior design”, “Duplicate selection and data extraction”, “Comprehensive search”, “Gray literature search”, “List of included and excluded studies”, “Characteristics of studies”, “Scientific quality assessed”, “Scientific quality in conclusions”, “Methods to combine studies”, “Publication bias assessment” and “Conflict of interest”) with four subscales (i.e., “yes”, “no”, “can’t answer” or “not applicable”).

### 2.5. Statistical Analysis

The outcome data were extracted from the GXT and expressed as mean ± SD. The meta-analysis was conducted using Review Manager software (RevMan, version 5.4). For each study, the standardized mean difference (SMD) with 95% confidence intervals (95% CI) was calculated to quantify changes in the performance variables, in which the mean or mean difference and corresponding SD were extracted from the pre- and post-intervention exercise tests and converted to the same unit. The effect of the K-LCHF diet on aerobic capacity and exercise performance was tested by inverse variance and a random effects model. The mean differences and 95% CI between studies were obtained through productive forest plots. SMD is the difference between the mean of the experiment group and the mean of the control group divided by the mean standard deviation, eliminating the effect of the “unit” so that the mean differences of different dimensions can be combined. SMD = 0 represents no difference, SMD > 0 represents more occurrence in the experimental group and SMD < 0 represents less occurrence in the experimental group. I^2^ statistics evaluated heterogeneity, with I^2^ values of 25%, 50% and 75% indicating low, medium and high heterogeneity, respectively. I^2^ > 50% indicated high heterogeneity between studies.

## 3. Results

### 3.1. Synopsis of Included Studies

**Figure 2:**
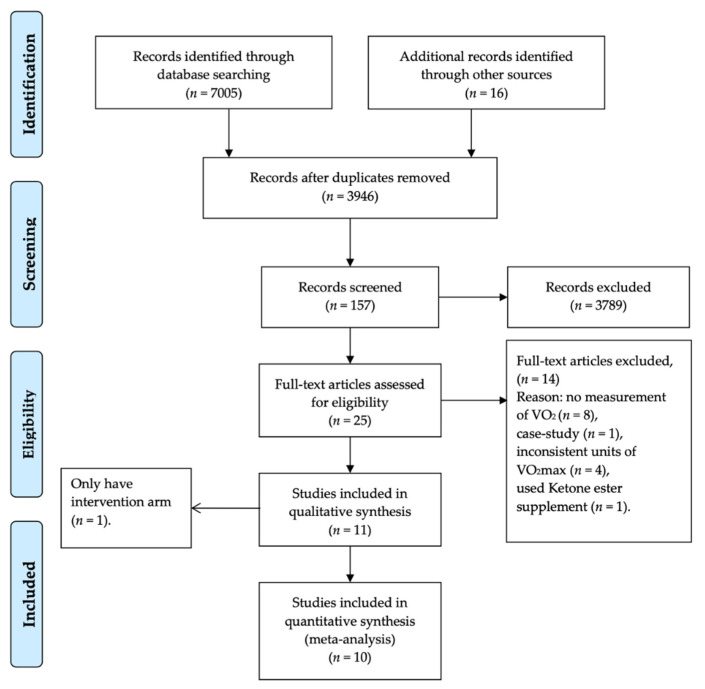
Flowchart for studies in the meta-analysis.

Of a total of 3946 non-duplicate studies, 3789 records were excluded by title and abstract inspection. The remaining 157 studies were assessed with full text, and 25 studies that met the inclusion criteria were selected. A further exclusion assessment was performed to remove the studies with no measurement of VO_2_max (*n* = 8), case-study design (*n* = 1), an inconsistent unit of VO_2_max (*n* = 4), use of a ketone ester supplement (*n* = 1) and having only an intervention arm (*n* = 1) (Figure 2). Finally, 10 articles were eligible for this report, including four crossover and six control studies.

### 3.2. Characteristics of Subjects

A total of 139 participants (5–24 healthy and/or trained adults in each study) in 10 studies [18,41,42,43,44,45,46,47,48,49] were included in this review. Except for the study of Burke et al. [18], all participants were male athletes. The four crossover studies had a small sample size (5–8 males) with older athletes (aged 30–50 years) compared with the other control trials. Six other control trials had a larger sample size (13–24 individuals) and young adults (aged 20–30 years, see Table 1).

### 3.3. Study Protocol

Ten studies investigated the effects of the K-LCHF diet on VO_2_max [18,41,42,43,44,45,46,47,48,49]. Among them, three studies investigated the effects of the K-LCHF diet on TTE [43,44,49]; eight studies investigated the effects of the K-LCHF diet on HRmax [18,39,40,41,42,43,44,46,47]; eight studies investigated the effects of the K-LCHF diet on RER [18,41,44,45,46,47,48,49]; and six studies investigated the effects of the K-LCHF diet on RPE [18,41,43,44,45,49]. The intervention protocol of the studies included in the review differed by dietary components, intervention duration, VO_2_max test type, other exercise tests and exercise session during intervention (Table 1). Most of the studies were conducted by using a treadmill test [18,41,42,43,46,48,49], while two studies were performed on a cycle ergometer [45,47]. All studies adopted the GXT to measure VO_2_max.

### 3.4. Intervention Implementation

In all studies, the intervention group received the K-LCHF diet, and the control group received a high-carbohydrate or habitual diet. The K-LCHF diet consisted of ≤10% CHO and ≥60% fat; all non-LCHF diets consisted of ≥40% CHO and ≤40% fat. Eight studies reported the daily energy intake (Table 1). Among those studies, the energy intake from the K-LCHF groups was in the range of 2000–4000 kcal/d; five studies reported a range of 3000–4000 kcal/d [18,41,42,48,49], while three studies reported a range of 2000–3000 kcal/d [44,45,46].

The durations of most interventions were two to six weeks [18,42,43,47,48,49]. One study had a short intervention of five days [41], while another had a relatively long intervention of 12 weeks [44].

### 3.5. Effect of K-LCHF Diet on VO_2_max during a GXT

**Figure 3:**
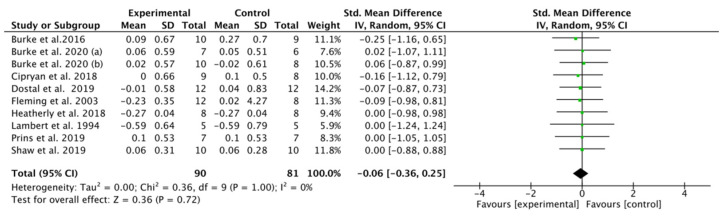
Effect of the ketogenic low-carbohydrate, high-fat (K-LCHF) diet on VO_2_max during graded exercise test (GXT); CI: confidence interval; SMD: standardized mean difference.

No significant overall difference in VO_2_max was found between the K-LCHF and HF/HD diets (SMD: −0.06, CI: −0.36, 0.25, *p* = 0.72). There was low heterogeneity in this analysis (I^2^ = 0%) (Figure 3). Only one study [42] reported a significantly increased VO_2_max within the group after the K-LCHF diet intervention.

### 3.6. Effect of K-LCHF Diet on TTE during a GXT

**Figure 4:**
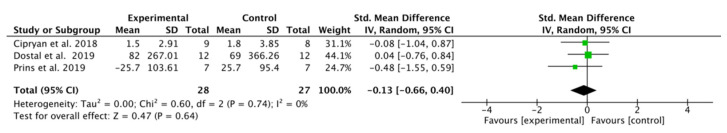
Effect of the ketogenic low-carbohydrate, high-fat (K-LCHF) diet on total time to exhaustion (TTE) during graded exercise test (GXT); CI: confidence interval; SMD: standardized mean difference.

TTE (minutes) was reported in three studies [43,44,48] involving 48 trained endurance athletes. No significant overall difference in TTE was found between the K-LCHF and HC/HD diet (SMD: −0.13, CI: −0.66, 0.40, *p* = 0.64), with an overall effect size of Z = 0.47 (Figure 4). There was low heterogeneity in this analysis (I^2^ = 0%).

### 3.7. Effect of K-LCHF Diet on Maximal Heart Rate (HRmax) during GXT

**Figure 5:**
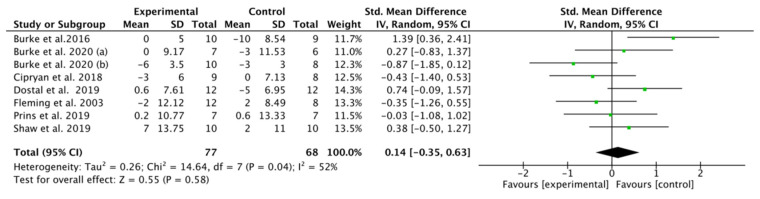
Effect of the ketogenic low-carbohydrate, high-fat (K-LCHF) diet on maximal heart rate (HRmax) during graded exercise test (GXT); CI: confidence interval; SMD: standardized mean difference.

HRmax was recorded in eight studies [18,41,42,43,44,45,46,48,49] involving 126 trained endurance athletes. No significant overall difference in HRmax was found between the K-LCHF and HC/HD diet (SMD: 0.14, CI: −0.35, 0.63, *p* = 0.58), with an overall effect size of Z = 0.55 (Figure 5). There was high heterogeneity in this analysis (I^2^ = 52%). Only one study [49] reported a significant increase in HRmax.

### 3.8. Effect of K-LCHF Diet on Maximal Respiratory Exchange Ratio (RER) during GXT

**Figure 6:**
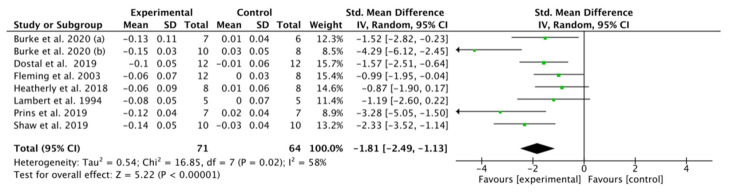
Effect of the ketogenic low-carbohydrate, high-fat (K-LCHF) diet on respiratory exchange ratio (RER) during graded exercise test (GXT); CI: confidence interval; SMD: standardized mean difference.

RER was presented in eight studies [18,41,44,45,46,47,48,49] involving 103 trained endurance athletes. A significant overall difference in RER was found between the K-LCHF and HC/HD diets (SMD: −1.81, CI: −2.49, –1.13, *p* < 0.00001), with an overall effect size of Z = 5.22 (Figure 6). There was high heterogeneity in this analysis (I^2^ = 58%). Those studies all showed a significant decrease in RER after the intervention.

### 3.9. Effect of K-LCHF Diet on RPE during GXT

**Figure 7:**
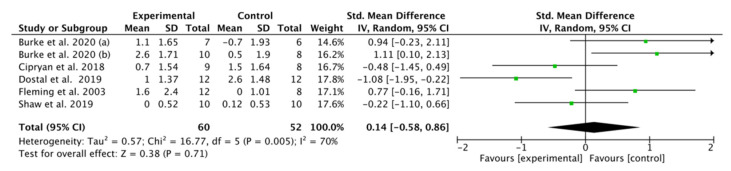
Effect of the ketogenic low-carbohydrate, high-fat (K-LCHF) diet on rating of perceived exertion (RPE) during graded exercise test (GXT); CI: confidence interval; SMD: standardize mean difference.

RPE was presented in six studies [18,41,43,44,45,49] involving 102 trained endurance athletes. No significant overall difference in RPE was found between the K-LCHF and HC/HD diets (SMD: 0.14, CI: −0.58, 0.86, *p* = 0.71), with an overall effect size of Z = 0.38 (Figure 7). There was high heterogeneity in this analysis (I^2^ = 70%). Only one study [45] showed a significant increase in RPE after the intervention.

## 4. Discussion

After reviewing the limited literature on the K-LCHF diet in endurance athletes, 10 eligible studies were included in the meta-analysis. Based on the outcomes of aerobic capacity, exercise performance and substrate oxidation in endurance athletes, we only found a significant effect of K-LCHF on RER, but not on VO_2_max, HRmax, TTE and RPE. This finding aligns with those of previous studies [18,41,43,44,45,46,47,48,49] that found that the K-LCHF diet had little effect on maximal aerobic capacity.

Under normal circumstances, glycogen stores in the liver and muscle cells need to break down to generate energy, and endogenous carbohydrates are stored mainly in the liver and muscle as a primary energy source in distance races [50,51,52,53,54,55]. In a previous study, Heatherly et al. [46] found that adaptation to a high-fat diet had a negative effect on VO_2_max owing to body mass reduction in middle-aged male runners. However, Helge et al. [56] found an increased VO_2_max after a fat-rich diet in untrained healthy males, and Phinney et al. found no change in endurance-trained athletes [4]. The K-LCHF diet might alter the maximal aerobic capacity through weight loss [45], but might not change VO_2_max as weight loss was not the primary objective in those studies, and is not the objective for endurance athletes generally. In another study, the K-LCHF diet was effective in extending some older athletes’ professional life by controlling or losing weight [9]. Another study analyzed gender differences after adopting the K-LCHF diet for four weeks and found a reduction in VO_2_max in women after the intervention, which was not observed in men [31]. We found that body mass was significantly decreased after the K-LCHF diet intervention in seven studies, but no significant changes in VO_2_max were observed. Of note, most studies have only reported on absolute VO_2_max. Absolute values indicate the total quantity of oxygen being used during exercise, while relative values indicate how aerobically fit someone is compared with their peers. In this report, two studies reported both absolute and relative VO_2_max, but neither showed a significant change after intervention [45,48]. Therefore, the interpretation of a K-LCHF diet strategy should be cautiously considered for athletic prowess in endurance sports if VO_2_max has not changed but body weight has decreased.

Moreover, no significant effect of K-LCHF diet was found on TTE. However, caution should be paid as only three studies with limited available data were used to examine the impact of the K-LCHF diet on the TTE. A reasonable explanation is that, during adaptation to the K-LCHF diet, individuals still had sufficient muscle glucose stores to sustain high-intensity exercise [57]. A high-fat diet significantly enhanced subsequent prolonged exercise at approximately 60% of VO_2_max, but, at the beginning of the workout, they only had 50% muscle glycogen content stored compared with the high-CHO-diet group [51]. In contrast, high-level athletes showed higher rates of fat oxidation, and their bodies utilized fat to replace part of the muscle glycogen for energy at a higher intensity [4]. This study also showed that after the body adapted to the K-LCHF diet, glycogen declined dramatically in muscle [4]. It remains to be studied whether long-term K-LCHF adaptation can restore the muscle glycogen to a comparable level [30]. Even though the ability to utilize fat was theoretically increased after the K-LCHF diet, no positive training effect was found on TTE, which may be related to the combination of diet and training.

Furthermore, no overall effect of the K-LCHF diet on HRmax was found. The potential neurological effect of a high-fat diet is that ketone adaptation increases the metabolic stress response during submaximal exercise. HR could be 7–9 bpm higher, potentially because of increased sympathetic nervous system activity [58]. A previous study suggested that the HR increases associated with obesity are caused by cardiac vagus nerve tension reduction [49]. Helge et al. [59] reported that subjects consuming a high-fat diet had significantly higher catecholamine and HR during submaximal exercise. Those changes may be related to changes in the autonomic nervous system activity at rest and in response to exercise after a short-term reduction in CHO intake (increased sympathetic and possibly decreased parasympathetic response) [59,60]. However, in our analysis, we did not find a significant effect of the K-LCHF diet on HRmax, which implies there is no evidence of a significant performance advantage after the K-LCHF diet (ketogenic or not). The ability to exercise at high intensity may be impaired by the K-LCHF diet.

The RER can indirectly indicate the ability of muscle to obtain energy [61]. A high RER indicates that carbohydrates are mainly used, while a low RER indicates that more fat is oxidized [61]. In our study, we found that the RER was significantly reduced after the adoption of the K-LCHF diet, indicating that more fat is involved in energy supply (Figure 6). In a study that simulated mountaineering after 4 h of cycling, eight out of nine participants improved their exercise ability during the climb after switching to a K-LCHF diet [62]. Throughout the study, five of the nine subjects enhanced their exercise ability by switching to the K-LCHF diet. Interestingly, comparing the high-CHO diet with the LCHF diet, RER improved. Exercise performance increased by an average of 375 s when climbing the mountain [62], indicating that a K-LCHF diet may be more advantageous to RER improvement in athletes. In addition, a study by Durkaleck-Michalski et al. [63] found males were more prone to switch macronutrient use from carbohydrate to more fat after the K-LCHF diet, reaching significance at the lower VO_2_ max levels. Conversely, females did not significantly decrease carbohydrate oxidation at any volume of VO_2_max. Our finding agrees with that of a previous report [62] suggesting that decreased RER after the K-LCHF diet may involve an energy supply drawing more from fat in endurance athletes.

RPE may decrease after a K-LCHF diet. Some studies have shown that ketones provide most of the fuel for the brain when CHO availability is insufficient and circulating β-HB concentrations are in the 1–5 mmol/L range (ketosis) [9,64,65]. However, not all K-LCHF diets may lead to ketosis [66]. Moreover, to a large extent, even minor dietary abnormalities can lead to an increase in the concentration of ketones in the body even though the diet is still LCHO [67]. After K-LCHF adaption, exercise may improve the brain center’s fatigue and cognitive function. This may be caused by the oxidation of β-HB, which provides a continued stable energy supply for the brain, delays the time of fatigue in the central nervous system and improves exercise performance [68,69,70]. However, no significant changes were found in RPE in this meta-analysis.

There was a large variation in the duration of the diet interventions among the included studies. However, when excluding the study by Burke et al. [41], with a five-day intervention, the results remained the same. Of note, the process of metabolic remodeling may initially take two weeks, with further adaptation in the following months to years [30]. In our report, most of the studies used interventions that were longer than two weeks. Even though we do not know the long-term adaptations, the results derived from these moderate lengths of interventions hint at the direction of the effect of K-LCHF on the aerobic capacity and exercise performance in endurance athletes.

### 4.1. Future Research

Different training strategies and study designs may explain the different impacts of the outcomes. Future studies should focus on developing an appropriate diet for endurance exercise and proposing guidelines for the intervention duration and intensity of training sessions for various groups of athletes. Furthermore, high-quality trials are required to prove the precise influence of different nutritional strategies.

### 4.2. Limitations

This study had some limitations. First, most of the studies only reported absolute VO_2_max, and TTE in the increment GXT was chosen as the primary outcome of exercise performance rather than the race time of real competitions. Second, only articles published in English were included, and gray literature and articles in other languages were not included. Third, we did not have CHO/fat oxidation data, so future study on analyses of CHO/fat oxidation would be useful. Finally, the subjects selected were all endurance athletes and almost all male, which is not representative of the general population. Moreover, there was no analysis of gender differences.

## 5. Conclusions

In summary, we found no significant overall effect of a ketogenic low-carbohydrate, high-fat diet on VO_2_max, HRmax, TTE and RPE, but a significant overall effect on RER. The K-LCHF diet did not lead to a positive change in aerobic capacity, possibly because the expected improvement was not achieved during the training period. Therefore, a K-LCHF diet is unlikely to change the aerobic capacity and exercise performance of endurance athletes, and there is a need to conduct high-quality intervention studies to assess the impact of different diet treatments for enhancing exercise performance in endurance athletes. 

## Figures and Tables

**Table 1:** Characteristics of included studies.

Citation, Year	Subjects	Age (Years)	Body Mass Change	Study Design	Dietary Components	Energy Intake	Intervention Duration	VO_2_max Test Type	Other Exercise Test	Exercise Session during the Intervention
Burke et al. 2017 [42]	19 highly competitive male race walkers K-LCHF: *n* = 10 HC: *n* = 9	K-LCHF: 28.3 ± 3.5 HC: 25.4 ± 4.0	K-LCHF: significantly reduction HC: no significant change	Control study	K-LCHF: 3.5% CHO 17% protein 78% fat HC: 60% CHO 16% protein 20% fat	K-LCHF: 3560 kcal/d HC: 3519 kcal/d	3 weeks	Graded economy test: Treadmill	10-km race; 20-km race	Race walking, resistance training and cross-training (running, cycling or swimming)
Burke et al. 2020 (a) [41]	13 male race walkers K-LCHF: *n* = 6 HC: *n* = 7	K-LCHF: 28.3 ± 2.7 HC: 32.7 ± 4.8	K-LCHF: significant reduction HC: no significant change	Parallel control study	K-LCHF: 4% CHO 16% protein 80% fat HC: 65% CHO 15% protein 20% fat	K-LCHF: 3766 ± 477 kcal/d HC: 3957 ± 726 kcal/d	5 days	Incremental testing: Treadmill	10-km race 25-km race	25–40 km walk, interval training session (8–12 × 1 km on a 6-min cycle), tempo hill session (14 km with ~450 m elevation gain). Low-intensity walking sessions (6–12 km each), and a strength training session
Burke et al. 2020 (b) [18]	18 highly competitive race walkers K-LCHF (M: 8; F: 2) HC (M: 5; F: 3)	K-LCHF: 29.9 ± 2.1 HC: 25.5 ± 3.6	K-LCHF: significant reduction HC: no significant change	Parallel control study	K-LCHF: 4% CHO 16% protein 78% fat HC: 65% CHO 15% protein 18% fat	K-LCHF: 3679 ± 382 kcal/d HC: 3345 ± 529 kcal/d	25 days	Graded exercise test (GXT): Treadmill	10-km race; 20-km race	Race walking, resistance training and cross-training (e.g., running, cycling or swimming)
Cipryan et al. 2018 [43]	17 moderately trained males K-LCHF: *n* = 9 HD: *n* = 8	K-LCHF: 23.8 ± 2.4 HD: 23.8 ± 1.8	K-LCHF: significant reduction HD: no significant change	Control study	K-LCHF: 8% CHO 29% protein 63% fat HD: 48% CHO 17% protein 35% fat	No report	4 weeks	Graded exercise test (GXT): Treadmill	No report	HIIT: 10-min warmup at 60% VO_2_max, followed by 5 high-intensity exercises consisting of 3 min at 100% VO_2_max (work to rest ratio, 2:1) Endurance-based running sessions or exercise (3–5 times a week)
Dostal et al. 2019 [44]	24 recreational trained runners K-LCHF: *n* = 12 HD: *n* = 12	K-LCHF: 25.3 ± 2.0 HD: 23.9 ± 3.8	K-LCHF: significant reduction HD: no significant change	Parallel control study	K-LCHF:8% CHO 23% protein 69% fat HD: 45% CHO 18% protein 37% fat	K-LCHF: 1960 ± 316 kcal/d HC: 1782 ± 412 kcal/d	12 weeks	Graded exercise test (GXT): Treadmill	No report	HIIT (sessions lasted approximately 40 min in total and consisted of a 4-min warmup followed by 5 × 6-min sets, separated by 2-min recovery), endurance exercise (e.g., running, cycling, sport games)
Fleming et al. 2003 [45]	20 non-highly trained men K-LCHF: *n* = 12 HC: *n* = 8	K-LCHF: 35 ± 13 HC: 36 ± 12	No report	Control study	K-LCHF: 8% CHO 30% protein 61% fat HC: 59% CHO 15% protein 25% fat	K-LCHF: 2235 ± 375 kcal/d HC: 1815 ± 195 kcal/d	6 weeks	Graded exercise test: Cycle ergometer	Wingate Sprint, Time ride	Walking, running, cycling and cross-training
Heatherly et al. 2018 [46]	8 trained runners	39.5 ± 9.9	K-LCHF: significant reduction HC: no significant change	Crossover study	K-LCHF: 7 ± 4% CHO 29 ± 9% protein 64 ± 9% fat HC: 43 ± 11% CHO 17 ± 8% protein 38 ± 7% fat	K-LCHF: 1886 ± 520 kcal/d HC: 2820 ± 955 kcal/d	3 weeks	Graded exercise test: Treadmill	50-min run in heat, 5-km time trial	No report
Lambert et al. 1994 [47]	5 endurance trained male cyclists	22.0 ± 1.80	No report	Crossover study	K-LCHF: 7% CHO 23% protein 70% fat HC: 74% CHO 14% protein 12% fat	No report	2 weeks	Progressive exercise test: Cycle ergometer	30-s Wingate test	No report
Prins et al. 2019 [48]	7 competitive recreational distance male runners	35.6 ± 8.4	No report	Randomized counterbalance crossover study	K-LCHF: 6.0 ± 1.3% CHO 25.1 ±1.5% protein 69 ± 2% fat HC: 56.4 ± 2.6% CHO 15.3 ±1.1% protein 28 ± 2% fat	K-LCHF: 2837 ± 251 kcal/d HC: 2947 ± 284 kcal/d	6 weeks	Graded exercise test: Treadmill	5-km time trial	Maintain usual training
Shaw et al. 2019 [49]	8 trained male endurance athletes	29.6 ± 5.1	K-LCHF: significant reduction HD: no significant change	Randomized crossover study	K-LCHF: 4.1 ± 0.8% CHO 18.2 ± 3.5% protein 78 ± 4% fat HD: 42.9 ± 7.8% CHO 18.6 ± 1.4% protein 39 ± 7% fat	K-LCHF: 3122 kcal/d HD: 3280 kcal/d	31 days	Graded metabolic test: Treadmill	Run to exhaustion trial	Running and cycling

Note: K-LCHF: ketogenic low-carbohydrate, high-fat diet group; HD: habitual diet group; HC: high-carbohydrate diet; CHO: carbohydrate; HIIT: high-intensity interval training.

## Data Availability

The data presented in this study are available on request from the corresponding author.

## Supplementary Materials

The following are available online at https://www.mdpi.com/article/10.3390/nu13082896/s1, Table S1: AMSTAR checklist items for each systematic review, Table S2: Search strategy used in each database.
