# Supplementary material for: The Effect of a Ketogenic Low-Carbohydrate, High-Fat Diet on Aerobic Capacity and Exercise Performance in Endurance Athletes: A Systematic Review and Meta-Analysis
Source: Nutrients. 2021 Aug 23;13(8):2896. doi: 10.3390/nu13082896 (PMC8400555; doi:10.3390/nu13082896)
Supplement: Supplementary file 1 [file nutrients-13-02896-s001.zip › Supplementary Materials.pdf]

Table S1. AMSTAR checklist items for each systematic review

AMSTAR Item / Systematic Review	Burke 2020.08	Burke 2020.06	Burke 2017	Cipyan 2018	Dostal 2019	Fleming 2003	Heathery 2018	Lambert 1994	Prins 2019	Shaw. 2019
1. A prior design	Yes	Yes	Yes	Yes	Yes	Yes	Yes	Yes	Yes	Yes
2.Duplicate selection and data extraction	Yes	Yes	Yes	Yes	Yes	Yes	Yes	Yes	Yes	Yes
3. Comprehensive search	Yes	Yes	Yes	Yes	Yes	Yes	Yes	Yes	Yes	Yes
4. Gray literature search	No	No	No	No	No	No	No	No	No	No
5. List if included and excluded studies	Yes	Yes	Yes	Yes	Yes	Yes	Yes	Yes	Yes	Yes
6. Characteristics of studies	Yes	Yes	Yes	Yes	Yes	Yes	Yes	Yes	Yes	Yes
7. Scientific quality assessed	Yes	Yes	Yes	Yes	Yes	Yes	Yes	Yes	Yes	Yes
8. Scientific quality in conclusions	Yes	Yes	Yes	Yes	Yes	Yes	Yes	Yes	Yes	Yes
9. Methods to combine studies	Yes	Yes	Yes	Yes	Yes	Yes	Yes	Yes	Yes	Yes
10. Publication bias assessment	No	No	No	No	No	No	No	No	No	No
11. Conflict of interest	Yes	Yes	Yes	Yes	Yes	Yes	Yes	Yes	Yes	Yes

Abbreviations: AMSTAR, A MeaSurement Tool to Assess Systematic Reviews; CA: cannot answer; NA: not applicable

Table S2. Search strategy used in each database

PubMed	#1low carbohydrate high fat diet [Title] OR ketogenic diet [Title]<br>#2 ((((exercise [Title] OR training [Title]) OR HIIT[Title]) OR VO2max [Title]) OR aerobic capacity [Title]) OR respiration [Title]<br>#3 #1 and #2
Web of Science	#1 TITLE: (low carbohydrate high fat diet) OR TITLE: (ketogenic diet)<br>#2 TITLE: (exercise) OR TITLE: (training) OR TITLE: (HIIT) OR TITLE: (VO2max) OR TITLE: (aerobic capacity) OR TITLE: (respiration)<br>#3 #1 and #2
Cochrane Library	#1 low carbohydrate high fat diet in Record Title OR ketogenic diet in Record Title<br>#2 exercise in Record Title OR training in Record Title OR HIIT in Record Title OR aerobic capacity in Record Title OR VO2max in Record Title OR respiration in Record Title<br>#3 #1 and #2
Pro Quest	#1 ti (low carbohydrate high fat diet) OR ti (ketogenic diet)<br>#2 ti (exercise) OR ti (training) OR ti (HIIT) OR ti (aerobic capacity) OR ti (VO2max) OR ti (respiration)<br>#3 #1 and #2
Science Direct	#1 low carbohydrate

	#2 ketogenic diet<br>#3 exercise<br>#4 training<br>#5 HIIT<br>#6 aerobic capacity<br>#7 VO_2max<br>#8 respiration<br>#9 #1 and #3<br>#10 #1 and #4<br>#11 #1 and #5<br>#12 #1 and #6<br>#13 #1 and #7<br>#14 #1 and #8<br>#15 #2 and #3<br>#16 #2 and #4<br>#17 #2 and #5<br>#18 #2 and #6<br>#19 #2 and #7<br>#20 #2 and #8
